# Urine Tumor DNA to Stratify the Risk of Recurrence in Patients Treated with Atezolizumab for Bacillus Calmette-Guérin–unresponsive Non–muscle-invasive Bladder Cancer

**DOI:** 10.1016/j.eururo.2025.03.023

**Published:** 2025-05-22

**Authors:** Marie-Pier St-Laurent, Parminder Singh, David J. McConkey, M. Scott Lucia, Vadim S. Koshkin, Kelly L. Stratton, Trinity J. Bivalacqua, Wassim Kassouf, Sima P. Porten, Rick Bangs, Melissa Plets, Ian M. Thompson, Joshua J. Meeks, Vincent M. Caruso, Ceressa T. Ward, Brian C. Mazzarella, Kevin G. Phillips, Vincent T. Bicocca, Trevor G. Levin, Seth P. Lerner, Peter C. Black

**Affiliations:** a University of British Columbia, Vancouver, BC, Canada; b Mayo Clinic Arizona, Phoenix, AZ, USA; c Johns Hopkins Greenberg Bladder Cancer Institute, Baltimore, MD, USA; d University of Colorado, Denver, CO, USA; e University of California San Francisco, Helen Diller Family Cancer Center, San Francisco, CA, USA; f University of Oklahoma, Oklahoma City, OK, USA; g University of Pennsylvania, Philadelphia, PA, USA; h McGill University Health Center, Montreal, QC, Canada; i Bladder Cancer Advocacy Network, Pittsford, NY, USA; j SWOG Statistics and Data Management Center, Seattle, WA, USA; k University of Texas Health Science Center at San Antonio, San Antonio, TX, USA; l Northwestern University, Feinberg School of Medicine, Chicago, IL, USA; m Convergent Genomics, Research and Development, San Francisco, CA, USA; n Baylor College of Medicine, Dan L Duncan Cancer Center, Houston, TX, USA

**Keywords:** Bladder cancer, Bacillus Calmette-Guérin unresponsive, Immune checkpoint inhibitor, Cell-free DNA, Urine biomarker

## Abstract

As new treatments for bacillus Calmette-Guérin (BCG)-unresponsive non–muscle-invasive bladder cancer (NMIBC) emerge, better methods are needed to guide therapeutic decisions. This study analyzed urine tumor DNA (utDNA) from patients treated with atezolizumab in the SWOG S1605 trial to determine whether utDNA profiling can stratify the risk of treatment failure. Urine samples were analyzed using the UroAmp assay at baseline and 3 mo from 89 and 77 patients, respectively. Only 13% of UroAmp-positive patients at baseline achieved a complete response at 6 mo compared with 71% of UroAmp-negative patients (*p* < 0.001). The 18-mo event-free survival (EFS) was significantly lower for UroAmp-positive patients at baseline (23%) than for UroAmp-negative patients (51%; hazard ratio [HR] 2.8, *p* < 0.001). Among patients with no clinical evidence of disease at 3 mo (*n* = 51), the 18-mo EFS was 38% for UroAmp-positive and 86% for UroAmp-negative (HR 3.5, *p* = 0.01) patients. These findings suggest that utDNA profiling at baseline and after 3 mo of treatment can help identify patients with BCG-unresponsive NMIBC who are less likely to benefit from systemic immunotherapy.

Treatment of bacillus Calmette-Guérin (BCG)-unresponsive high-risk non–muscle-invasive bladder cancer (NMIBC) remains challenging despite emerging therapies. Radical cystectomy (RC) is the standard of care, but it carries a significant risk of complications as well as the life-altering impact of urinary diversion [1]. As a result, many patients choose bladder-preserving therapies despite the higher risks of recurrence, progression, and cancer-related mortality.

As multiple new bladder-preserving therapies enter clinical practice, there is an increasing need for effective biomarkers to predict and monitor treatment efficacy [2–4]. Current methods primarily rely on cystoscopy and cytology, both of which have limitations and do not inform prognosis or possible early treatment change. Profiling of urine tumor DNA (utDNA) has emerged as a tool to detect residual disease and stratify patients based on recurrence risk. UroAmp MRD, a multigene assay measuring genomic alterations in urine, was shown to stratify risk in patients with BCG-naïve NMIBC [5]. This study investigates the association of UroAmp with clinical outcome in patients with BCG-unresponsive NMIBC treated with atezolizumab, an anti–PD-L1 monoclonal antibody [6].

The SWOG S1605 study (NCT02844816) was a single-arm, phase 2 trial assessing atezolizumab in patients with BCG-unresponsive high-risk NMIBC [6]. Complete resection of a visible tumor was required prior to enrollment. The primary endpoints were a complete response (CR) confirmed by a mandatory biopsy at 6 mo for patients with carcinoma in situ (CIS) at study entry and event-free survival (EFS) at 18 mo for all patients. The trial followed the Declaration of Helsinki and good clinical practice guidelines under the NCTN, led by SWOG, and was approved by the National Cancer Institute central institutional review board. All patients provided written informed consent.

UroAmp testing was performed, as described previously [7], on urine samples collected at baseline and 3 mo. The assay quantified disease classification and genomic disease burden by analyzing specific somatic mutations, copynumber variations, and aneuploidy patterns in utDNA [8]. Additional information on study design, methods, and genomic analysis is detailed in the Supplementary material.

UroAmp results were generated in a blinded fashion and provided to SWOG statisticians to correlate with clinical outcomes. Cox proportional hazards regression, adjusted for baseline CIS status, was performed to estimate EFS hazard ratios (HRs) for UroAmp-positive versus UroAmpnegative patients. Kaplan-Meier estimates were calculated to compare 18-mo EFS. Two-sided *p* values of <0.05 were considered statistically significant. These analyses were conducted in SAS 9.4 and exploratory analyses were conducted in Python using the lifelines, SciPy, and statsmodels package.

After completion of the blinded analysis, unblinding revealed misclassification of four patients. A revised analysis with reclassification of these four samples was performed and is reported in the Supplementary material.

Of the 172 registered patients, 129 were eligible and included in the efficacy analysis of SWOG S1605. Among these, 98 patients provided adequate urine samples for UroAmp testing, including 89 with a baseline sample and 77 with a 3-mo sample (Supplementary Fig. 1). The demographics and tumor characteristics are detailed in Supplementary Table 1.

At baseline, UroAmp was positive in 61/89 (69%) patients, including 38/52 (73%) with CIS ± Ta/T1 and 23/37 (62%) with Ta/T1 tumors after prior resection of all visible tumors (Fig. 1A). The 6-mo CR rate in UroAmp-positive CIS patients was 13% (5/38), compared with 71% (10/14) in UroAmp-negative patients (*p* < 0.001; Fig. 1B). Among UroAmp-positive Ta/T1 patients at baseline (*n* = 23), the 18-mo EFS rate was 43%, compared with 71% for UroAmp-negative patients (*n* = 14; HR 3.2, 95% confidence interval [CI]: [1.2, 8.4]; *p* = 0.018; Supplementary Fig. 2). The 18-mo EFS in the overall cohort was 23% for UroAmp-positive patients versus 51% for UroAmp-negative patients (HR 2.8, 95% CI: [1.6, 5.1], *p* < 0.001; Fig. 1C). Molecular profiling revealed considerable variability in mutation patterns among patients (Supplementary Fig. 3A–C and 4A–C). A comparison between baseline UroAmp and cytology is provided in Supplementary Table 2.

UroAmp also stratified subsequent CR and EFS rates in patients who remained event free at 3 mo (*n* = 51; Fig. 2A). The 6-mo CR rate in UroAmp-positive CIS patients was 47% (7/15), compared with 100% (2/2) in UroAmpnegative patients. In UroAmp-positive Ta/T1 patients (*n* = 22), the 18-mo EFS was 47%, compared with 83% for UroAmp-negative patients (*n* = 12; HR 3.2, 95% CI: [1.1, 9.7], *p* = 0.039). The 18-mo EFS in the overall cohort was 38% in UroAmp-positive (*n* = 37) and 86% in UroAmp-negative patients (*n* = 14; HR 3.5, 95% CI: [1.3, 9.1], *p* = 0.012, adjusted for CIS; Fig. 2B). The correlation between 3-mo cytology and UroAmp and the 3- and 6-mo clinical outcome is available in the Supplementary material. Accuracy of the test with 3-mo outcome was higher with cytology (72% vs 44%), but UroAmp had sensitivity and a negative predictive value of 100%. When comparing with clinical outcome at 6 mo, UroAmp had higher accuracy than cytology (69% vs 55%; Supplementary Tables 3 and 4).

Management of patients with BCG-unresponsive NMIBC is hindered by a lack of biomarkers to guide treatment selection and monitor outcome [1,9]. In this study, UroAmp was able to stratify CR and EFS rates in patients treated with atezolizumab. The strong baseline prognostic impact of utDNA burden is consistent with plasma circulating tumor DNA in patients with more advanced disease [10,11]. All patients were treated with atezolizumab, so we can draw no conclusions on the predictive versus prognostic capacity of utDNA in this context. We would postulate that the results would be applicable to patients treated with other PD-(L)1 inhibitors. Overall, our results and those of others [12] suggest that UroAmp may be able to identify patients unlikely to benefit from specific bladder-preserving treatments, allowing for early consideration of alternative therapies including RC.

The study’s limitations include small sample size and a lack of standardized biopsy protocols at 6 mo in the CIS cohort. Urine pellets were collected at many sites without the addition of a buffer to prevent white blood cell lysis and without buffy coat control, which limits the ability to control for potential clonal hematopoiesis. Misclassification of the clinical or genomic status in four patients could undermine confidence in the study results. The 60-gene panel does not analyze all known bladder cancer mutations.

Future trials should consider studying utDNA as a tool for refining inclusion criteria, balancing trial arms, stratifying for treatment escalation or de-escalation, and potentially utilizing on-treatment molecular response as an intermediate endpoint.

UroAmp effectively stratified CR and EFS rates in patients with BCG-unresponsive NMIBC treated with atezolizumab. These findings support the integration of utDNA profiling into further trial designs to help guide treatment and surveillance.

This trial was presented previously at ASCO GU 2024.

## Supplementary Material

1

Supplementary data to this article can be found online at https://doi.org/10.1016/j.eururo.2025.03.023.

## Figures and Tables

**Fig. 1 –:**
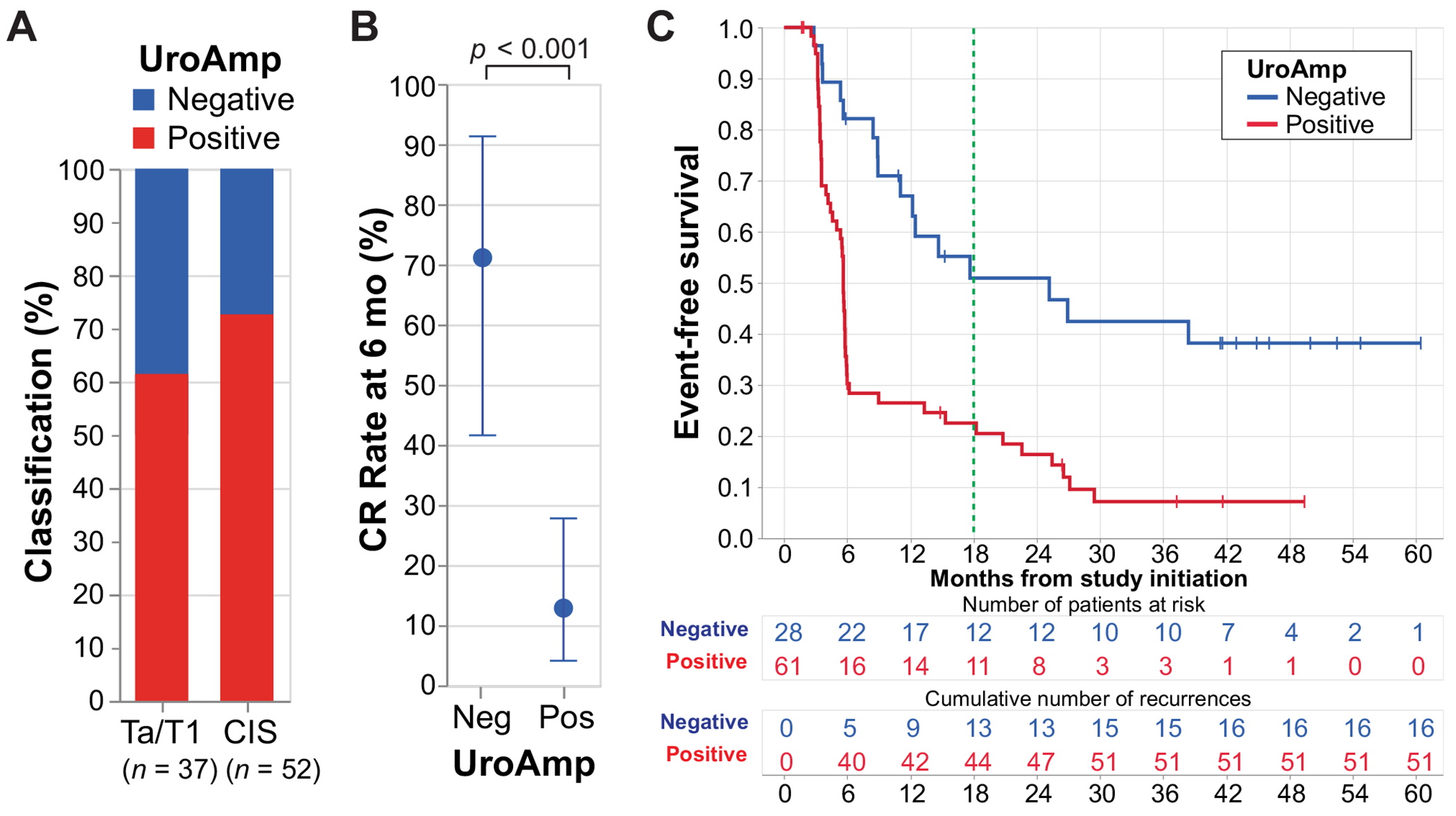
Baseline UroAmp stratifies the rates of complete response and event-free survival. Profiling of utDNA was performed in non–muscle-invasive bladder cancer patients following transurethral resection of bladder tumor and prior to treatment initiation. (A) Proportion of patients classified as UroAmp negative (*n* = 28) and positive (*n* = 61) according to carcinoma in situ (CIS) status at study enrollment. (B) Six-month complete response (CR) rate with 95% confidence interval for CIS patients classified as UroAmp negative (*n* = 14) or positive (*n* = 38). Statistical significance was determined by the Cochran-Mantel-Haenszel test. (C) Event-free survival by UroAmp status, negative versus positive. Hazard ratios were determined by Cox proportional hazards models with adjustment for CIS at baseline. Time 0 is the date of study registration. Neg = negative; Pos = positive; utDNA = urine tumor DNA.

**Fig. 2 –:**
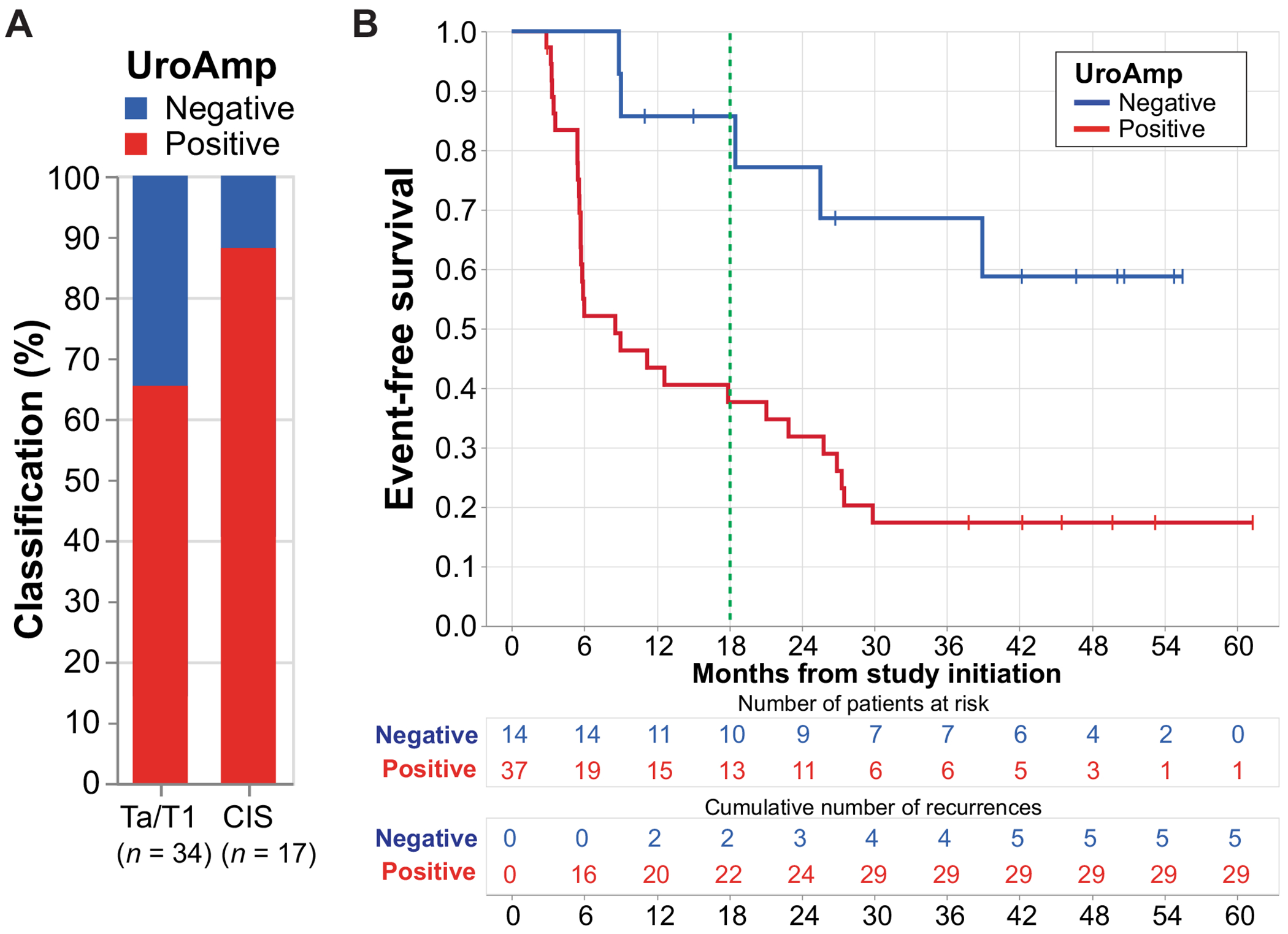
UroAmp after 3 mo of atezolizumab treatment predicts subsequent event-free survival. Urinary tumor DNA (utDNA) profiling was performed after four cycles of atezolizumab at the first surveillance time point (3 mo) in patients who were not found to have a clinical recurrence (cystoscopy ± biopsy). (A) Proportion of patients classified as UroAmp negative (*n* = 14) and positive (*n* = 37) according to carcinoma in situ (CIS) status at study enrollment. (B) Event-free survival stratified by UroAmp status; Cox proportional hazard ratio 3.5 (*p* = 0.012, 95% CI [1.3, 9.1]), adjusted for CIS status at baseline. CI = confidence interval.

## ADVANCING PRACTICE

### What does this study add?

This study demonstrates that urine tumor DNA profiling can be used to stratify the risk of recurrence in patients after treatment with atezolizumab for bacillus Calmette-Guérin–unresponsive non–muscle-invasive bladder cancer.

### Clinical Relevance

We have a critical need to identify accurate biomarkers to further hone risk stratification and personalize therapeutic decisions. This is especially critical in the setting of high risk BCG-unresponsive nonmuscle invasive bladder cancer (NMIBC) where recurrence and progression in the setting of an ineffective bladder therapy could have significant oncologic implications. The authors present a secondary analysis of the SWOG 1605 study, a single arm phase 2 clinical trial evaluating the efficacy of intravenous atezolizumab for the treatment of BCG-unresponsive nonmuscle invasive bladder cancer, in which they demonstrated that baseline and 3-month urine tumor DNA (utDNA) was predictive of the complete response rate at 6-months and event-free survival at 18-months following treatment with atezolizumab, respectively. The results of this study support further evaluation of utDNA as a predictive biomarker for the selection of optimal patients for sequential lines of bladder-sparing therapies for patients with high risk, BCG-unresponsive NMIBC. Associate Editor: Sarah P Psutka.

### Patient Summary

We have assessed the ability of a novel urine test to stratify the risk of response to treatment with the immunotherapy drug atezolizumab. In patients with high-risk non–muscle-invasive bladder cancer that recurred after prior bacillus Calmette-Guérin treatment, we found that the urine-derived tumor DNA test (UroAmp) can identify which patients are at a high risk of cancer recurrence before and during the treatment with atezolizumab. Patients with positive urine tumor DNA were less likely to respond to treatment and had a higher chance of cancer recurrence. This test could help doctors and patients make a better decision about which treatments are most likely to work in individual cases.
