# Supplementary material for: Urine Tumor DNA to Stratify the Risk of Recurrence in Patients Treated with Atezolizumab for Bacillus Calmette-Guérin–unresponsive Non–muscle-invasive Bladder Cancer
Source: Eur Urol. Author manuscript; Available in PMC 2026 Jun 5. (PMC13237821; doi:10.1016/j.eururo.2025.03.023)
Supplement: 1 [file NIHMS2174402-supplement-1.docx]

**Supplementary material**

**Study design and patients**

SWOG S1605 was an open-label, single-arm, phase 2 clinical trial conducted across 68 National Clinical Trials Network (NCTN) group sites in the USA and Canada between February 7, 2017 and July 5, 2019. The primary trial results including details of the trial design and the trial protocol itself have been published previously [1]. The trial is registered on ClinicalTrials.gov as NCT02844816. Patients received 1200 mg of atezolizumab intravenously every 3 wk for up to 17 cycles (51 wk) in the absence of disease recurrence or unacceptable toxicity. The trial was conducted according to the Declaration of Helsinki and in compliance with good clinical practice guidelines through the NCTN under the leadership of SWOG, and received approval from the National Cancer Institute central institutional review board. Patients provided written, informed consent.

***Patients***

Eligible participants had histologically confirmed urothelial carcinoma (UC) of the bladder that met the criteria for bacillus Calmette-Guérin (BCG)-unresponsive high-risk non–muscle-invasive bladder cancer (NMIBC) according to the guidance statement of the Food and Drug Administration (FDA) [2]. BCG-unresponsive NMIBC was defined as carcinoma in situ (CIS; ±Ta/T1 tumor) recurring within 12 mo or high-grade (HG) Ta/T1 tumor (without CIS) recurring within 6 mo of the last dose of adequate BCG. Adequate BCG was defined as at least induction BCG (five or more doses) and the first round of maintenance (two or more doses) or a second induction course of BCG (two or more doses) for patients with CIS/Ta disease at the time of recurrence, and at least induction BCG (five or more doses) for patients with T1 tumors at the time of recurrence. Patients were stratified as CIS ± Ta/T1 tumors (CIS cohort) versus HG Ta/T1 tumors without CIS (papillary-only cohort). Ta and/or T1 tumors required visual complete transurethral resection of bladder tumor (TURBT), and patients with T1 tumors required restaging TURBT, with the presence of muscularis propria in at least one of the two TURBT procedures. The most recent TURBT had to be within 60 d of registration, and a cystoscopy confirming the absence of visible papillary disease was required within 21 d.

***Patient monitoring***

Patients were monitored using cystoscopy and cytology every 3 mo for 24 mo, and then every 6 mo for the following 36 mo. All patients with CIS at study entry underwent mandatory biopsy at 6 mo. Treatment and surveillance after HG recurrence were left to the discretion of the investigator.

***Urinary comprehensive genomic profiling***

Urine samples were collected at baseline (immediately prior to induction with atezolizumab) and again at 3 mo (just prior to the fifth cycle of therapy). Samples were spun and frozen without preservatives on site. All collected urine samples were stored at –80°C. Samples were transferred to the sponsor (Convergent Genomics, San Francisco, CA, USA) in a blinded fashion, ensuring that the clinical status of samples remain unknown to the sponsor’s laboratory or data analysis personnel during sample processing. Profiling of urinary tumor DNA (utDNA) was performed with the Clinical Laboratory Improvement Amendments (CLIA)-validated UroAmp platform at the College of American Pathologists (CAP)-accredited Convergent Genomics laboratory.

UroAmp uses next-generation sequencing to detect single-nucleotide variants, small insertion-deletions, targeted gene-level copy-number variants, microsatellite instability, and copy-neutral loss of heterozygosity across a 60-gene panel, as well as whole-genome aneuploidy. Mutation profiles serve as input features to algorithms that predict tumor presence, molecular grade, and recurrence risk [3]. UroAmp recurrence risk assignments are calculated independently of any clinical features. UroAmp testing methodology was performed as described previously [4].

***Clinical outcomes, UroAmp status, and statistical analysis***

The primary endpoints of the trial were complete response (CR) rate at 6 mo in CIS patients and event-free survival (EFS) in all eligible patients, including interest in EFS rate at 18 mo after registration. The 6-mo CR for the CIS cohort was defined as absence of HG UC on mandatory bladder biopsy and urine cytology, and absence of an upper tract or urethral second primary tumor or more advanced UC. An event was defined as the first occurrence of biopsy-proven HG UC of the bladder, urethra, or upper tract; clinical evidence of metastasis; or death from UC. Time to event was calculated from the date of study registration for the baseline assessment and from the date of urine collection for the 3-mo time point. Patients who died of causes other than UC and patients last known to be alive and disease free were censored at the date of last disease assessment.

UroAmp status and genomic disease burden (GDB) were determined from both baseline and 3-mo samples. GDB is a percentile ranking based on a composite score that considers the number of mutations, mutation type, and allele frequency [3]. Two established algorithms were tested:

1. UroAmp MRD algorithm—classification as “positive” or “negative.” Algorithm training and validation were described previously [3].
2. UroAmp GDB-enhanced algorithm—incorporates a GDB threshold (≥42) into the previously established UroAmp MRD algorithm. This threshold was determined by a subanalysis of BCG-treated surveillance patients from previous training and validation studies [3,5]. A gain/drop of ≥10 was established empirically from reproducibility studies where the same samples were analyzed multiple times, and technical variation in GDB was always found to be <10 points. Therefore, changes in GDB of ≥10 are considered to originate predominately from biological sources rather than technical variation. The experimental GDB-enhanced algorithm results are presented only in Supplementary Fig. 6.

Paired longitudinal samples were used to assess molecular response using baseline (pretreatment) GDB and change in GDB (after treatment minus before treatment).

Blinded results were reported to SWOG statisticians prior to the release of clinical and genomic data. Cox proportional hazards regression, adjusted for baseline CIS status, was performed to estimate EFS hazard ratios (HRs) for UroAmp-positive versus UroAmp-negative patients. Kaplan-Meier point estimates were calculated to compare 18-mo EFS of UroAmp-positive versus UroAmp-negative patients. Two-sided *p* values of <0.05 were considered statistically significant. The correlation between urine cytology and UroAmp was assessed using diagnostic performance metrics, confidence interval (CI) using exact binomial method, and Cohen's kappa to quantify the level of agreement between two measures. Main analyses were conducted in SAS 9.4. All other exploratory statistical analyses were performed in Python using the lifelines, SciPy, and statsmodels packages [6–8].

**Results**

The median follow-up time for alive patients without a recurrence was 42 mo from study registration. As reported previously in SWOG S1605, the 6-mo CR rate in the CIS ± Ta/T1 cohort was 27% [1]. The 18-mo EFS rate was 49% in the Ta/T1 cohort and 33% in their overall cohort. In the UroAmp subset, the 6-mo CR rate in the CIS ± Ta/T1 cohort was 29%, and the 18-mo EFS rate Kaplan-Meier (without stratification) for the UroAmp subset with baseline sample (*n* = 89) was 32% (95% CI: 23.2–43.8).

UroAmp was negative at 3 mo in 14/51 patients (12/34 papillary only Ta/T1). Only four patients converted from positive to negative, eight were UroAmp negative at both time points, and two did not have a baseline sample available for an analysis.

***Comparison with urine cytology***

At baseline, 11 patients had a positive cytology result and 78 patients had negative or atypical cytology. Supplementary Table 2 summarizes UroAmp results compared with cytology. There was minimal agreement between UroAmp and cytology (Cohen's kappa = 0.086 [*z* = 1.71], *p* = 0.088).

At the 3-mo time point, 75 patients had both cytology and UroAmp results available. Among these patients, nine had positive cytology and 66 had negative or atypical cytology. Supplementary Table 3 summarizes UroAmp compared with cytology results at 3 mo. There was a nonsignificant minimal agreement between UroAmp and cytology (Cohen's kappa = 0.0293 [*z* = 0.71], *p* = 0.48).

The performance characteristics of UroAmp and cytology collected at the 3-mo time point to detect HG UC at the same assessment were measured (Supplementary Table 4). Cytology had 17% sensitivity, 90% specificity, 33% positive predictive value (PPV), and 77% negative predictive value (NPV) with 72% accuracy. UroAmp had 100% sensitivity, 26% specificity, 30% PPV, and 100% NPV with 44% accuracy. The performance of both tests to correlate with the presence of recurrence at the next assessment (6 mo from study enrollment) was also tested. Cytology had sensitivity of 18%, specificity of 94%, PPV of 78%, and NPV of 52% with reduced accuracy of 55%. UroAmp had 97% sensitivity, 39% specificity, 63% PPV, 93% NPV, and improved accuracy of 69% (Supplementary Table 4).

***Molecular characterization***

We compared the molecular characteristics of the urine from the baseline sample of patients with CIS (*n* = 56) and Ta/T1 tumors (*n* = 42). Mutations in *RB1* (undefined odds ratio [OR], *p* = 0.061), *ARID1A* (OR 10.1, *p* = 0.009), *TP53* (OR 3.8, *p* = 0.061), and *TERT* (OR 3.1, *p* = 0.061) were significantly associated with CIS (Supplementary Fig. 5A). Copy-number amplifications of *NIT1* and *SOX4* and single-nucleotide mutations in *ERBB3* were enriched in CIS patients, but not at a statistically significant level.

The complete panel of mutations identified in utDNA is presented in an oncopanel (Supplementary Fig. 3B). Data on the most prevalent variants at 3-mo follow-up samples are also provided for all patients and patients who recurred (Supplementary Fig. 4B and 4C). A comparative analysis with baseline samples demonstrated a consistent pattern in the types and frequencies of gene alterations before and after therapy, with a noted increase in TERT, TP53, and ZFP36L1 variants after treatment.

***Exploratory GDB-enhanced classification***

Using baseline samples, the experimental GDB-enhanced algorithm classified patients identically to the validated UroAmp algorithm, with no change in HR. However, on the 3-mo samples, the experimental GDB-enhanced algorithm reclassified one patient from negative to positive (HR 4.3, 95% CI: [1.5, 12.4]; *p* = 0.007; adjusted for CIS; Supplementary Fig. 6).

***Revised study analysis***

Following the release of clinical and genomic data to coauthors, clinical and genomic data analyses performed in the support of this study revealed four patient misclassifications in the blinded analyses. All results from the main manuscript are from blinded analyses, including EFS and CR analyses. Revised EFS and CR analyses with corrected misclassifications are presented here. Additionally, the association of GDB score with EFS was analyzed across a total of 98 patients independent of the UroAmp algorithm.

Clinical event status was revised for two patients with CIS after 3 mo of atezolizumab treatment who were considered event free as per the initial SWOG S1605 definition [1], because patients could continue with study treatment up to 6 mo if found to have HG Ta or CIS at 3 mo. However, in this analysis, an event is defined as any HG UC bladder biopsy at 3 mo. Their status was rectified here to reflect being event positive. UroAmp status was revised in two additional patients after a quality control review, as mandated for CLIA reporting. One patient was reclassified from UroAmp positive to negative after removal of an *ELF3* mutation identified at 50% VAF that was determined to be a rare germline single-nucleotide polymorphism (1-202012049-G-A) enriched in people of non-Finnish European ancestry [9]. The second patient was classified as UroAmp positive, but had a GDB of 0 and no reportable mutations. This patient was reclassified as UroAmp negative following the identification of filtering errors in variant annotation software that were corrected subsequently. The errors would have been corrected within standard operating protocols in the commercial assay, as dictated by the CLIA quality control review, but manual inspection of the results did not occur as part of the research sample pipeline prior to unblinding.

***Corrected analysis and UroAmp recurrence prediction at baseline***

The revised analysis identified 59 of 89 (66%) patients as UroAmp positive, including 38 of 52 (73%) CIS patients and 21 of 37 (57%) Ta/T1 patients. Among UroAmp-positive Ta/T1 patients (*n* = 21), the 18-mo EFS rate was 43%, compared with the rate of 75% for UroAmp-negative patients (*n* = 16). UroAmp-positive patients had 18- and 36-mo EFS rated of 20% and 5%, compared with 54% and 46% for negative patients, respectively (all-follow-up HR 3.2, 95% CI: [1.8, 5.8]; *p* < 0.001, adjusted for presence of CIS; Supplementary Fig. 7A). The experimental GDB-enhanced algorithm classified patients identically to the validated UroAmp algorithm. The presence of CIS was the strongest clinical predictor of events (all-follow-up HR 2.0; 95% CI: [1.2, 3.4]; *p* = 0.008) adjusted for UroAmp status (Supplementary Fig. 7B).

***Corrected analysis and UroAmp recurrence prediction at 3 mo***

The revised analysis was performed in eligible patients who provided adequate urine sample prior to re-evaluation at 3 mo and were found to be event free at this time point. UroAmp was positive at 3 mo in 36 of these 51 (71%) patients, including 15 of 17 (88%) CIS patients and 21 of 34 (62%) Ta/T1 patients. Among UroAmp-positive Ta/T1 patients (*n* = 21), the 18-mo EFS rate was 44%, compared with the rate of 85% for UroAmp-negative patients (*n* = 13; all-follow-up HR 4.0, 95% CI: [1.3, 12.1]; *p* = 0.015). Based on an analysis of 3-mo urine samples in patients determined to be event free at this time point, the 18-mo EFS was 36% in UroAmp-positive patients, compared with 87% in UroAmp-negative patients (HR 4.1, 95% CI: [1.5, 10.9]; *p* = 0.005; Supplementary Fig. 8). The experimental GDB-enhanced algorithm reclassified one patient from negative to positive (all-follow-up HR 5.1, 95% CI: [1.8, 14.8]; *p* = 0.003; adjusted for CIS; Supplementary Fig. 9).

***Molecular response to treatment***

Patients with paired baseline and 3-mo samples (*n* = 68) were examined for a molecular response to treatment using the baseline GDB and the change in GDB between the two time points. Cox survival modeling of median EFS estimates based on longitudinal GDB scores demonstrated the relationship between baseline GDB and GDB change in response to atezolizumab (Supplementary Fig. 10). For example, a patient with a partial response, who began therapy with a GDB of 60 and experienced a GDB drop by 67%, could expect the same median EFS as an expanding patient whose GDB started at 20 but doubled on therapy (Supplementary Fig. 10). For patients beginning therapy with the highest GDB (>80), even the best response (GDB reduction of 60) was insufficient to achieve median EFS of 12 mo.

***Expanded molecular analysis of CIS versus papillary-only patients***

An expanded analysis comparing genomic profiles of CIS patients with those of papillary-only patients was conducted, which included an additional 32 patients who were initially enrolled in the trial but later determined not to meet the FDA definition of BCG-unresponsive patients. An enrichment analysis of this expanded cohort (*n* = 121) confirmed the findings from the drug-eligible cohort and additionally provided statistical significance to the previously observed enrichment in CIS patients of NIT1 and SOX4 copy-number amplifications (Supplementary Fig. 5B).

**Supplementary Fig. 1 – Standards for Reporting of Diagnostic Accuracy Studies (STARD) diagram detailing specimen usage in this study. SWOG S1605 exclusion criteria are detailed in the study by Black et al (2023) [1].**

**Supplementary Table 1 – Baseline characteristics of eligible patients included in the primary efficacy analyses and the UroAmp molecular analyses**

|  | S1605 primary analysis [1]  (*N* = 129) | UroAmp molecular analysis  (*N* = 98) |
| --- | --- | --- |
| Median age (range) | 74.0 (38.3, 97.7) | 74.7 (46.6, 97.7) |
| Race, *N* (%) |  |  |
| White | 119 (92) | 93 (95) |
| Non-White | 10 (8) | 5 (5) |
| Male, *N* (%) | 108 (84) | 81 (83) |
| Baseline stage, *N* (%) |  |  |
| CIS only | 43 (33) | 30 (31) |
| CIS + Ta/T1 | 31 (24) | 26 (27) |
| Ta only | 30 (23) | 23 (24) |
| T1 ± Ta | 25 (20) | 19 (19) |

CIS = carcinoma in situ.


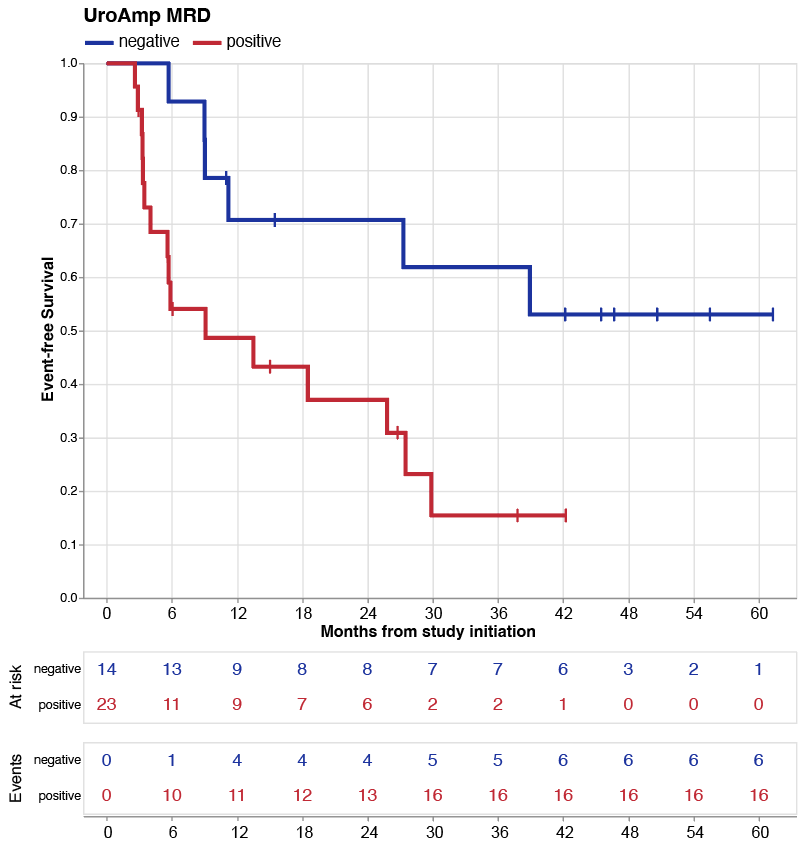


**Supplementary Fig. 2 – Event-free survival by UroAmp status (negative vs positive) at baseline for Ta/T1 patients without concomitant CIS. Hazard ratios were determined by Cox proportional hazards models (HR 3.2, *p* = 0.018, 95% CI: [1.2, 8.4]). Time 0 is the date of study registration. CI = confidence interval; CIS = carcinoma in situ; HR = hazard ratio.**


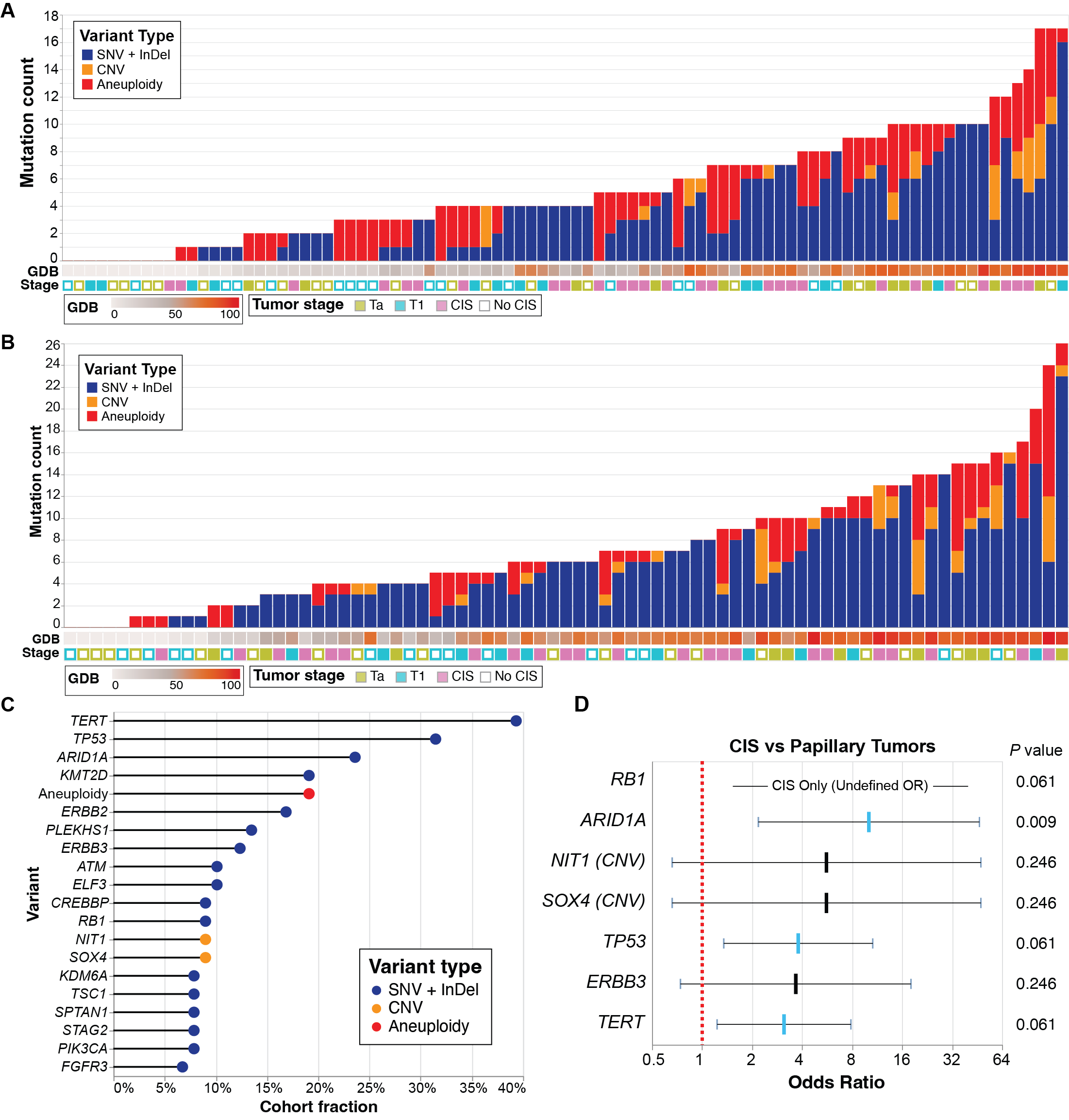

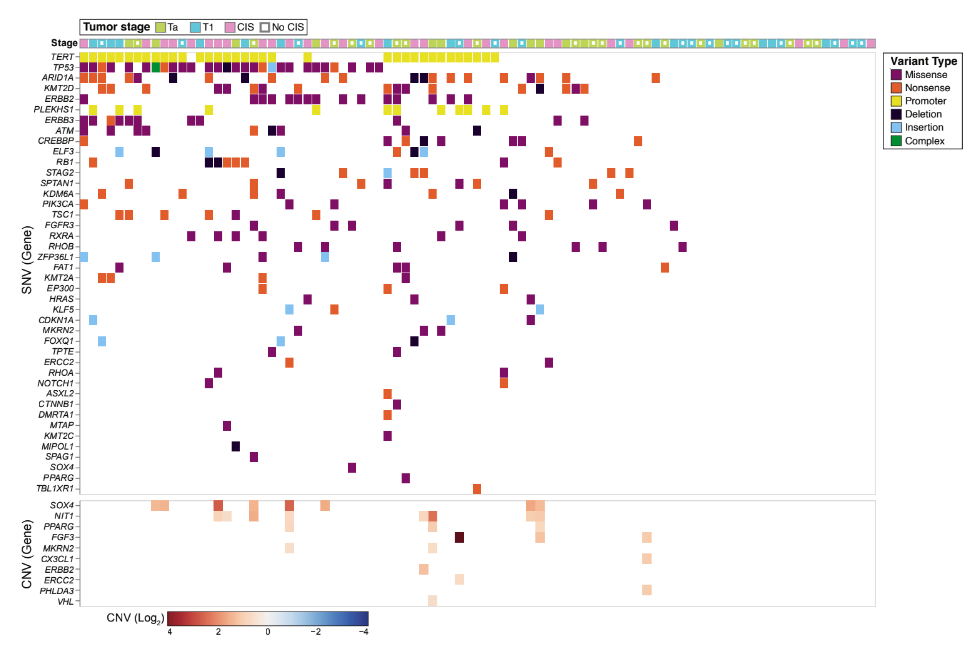

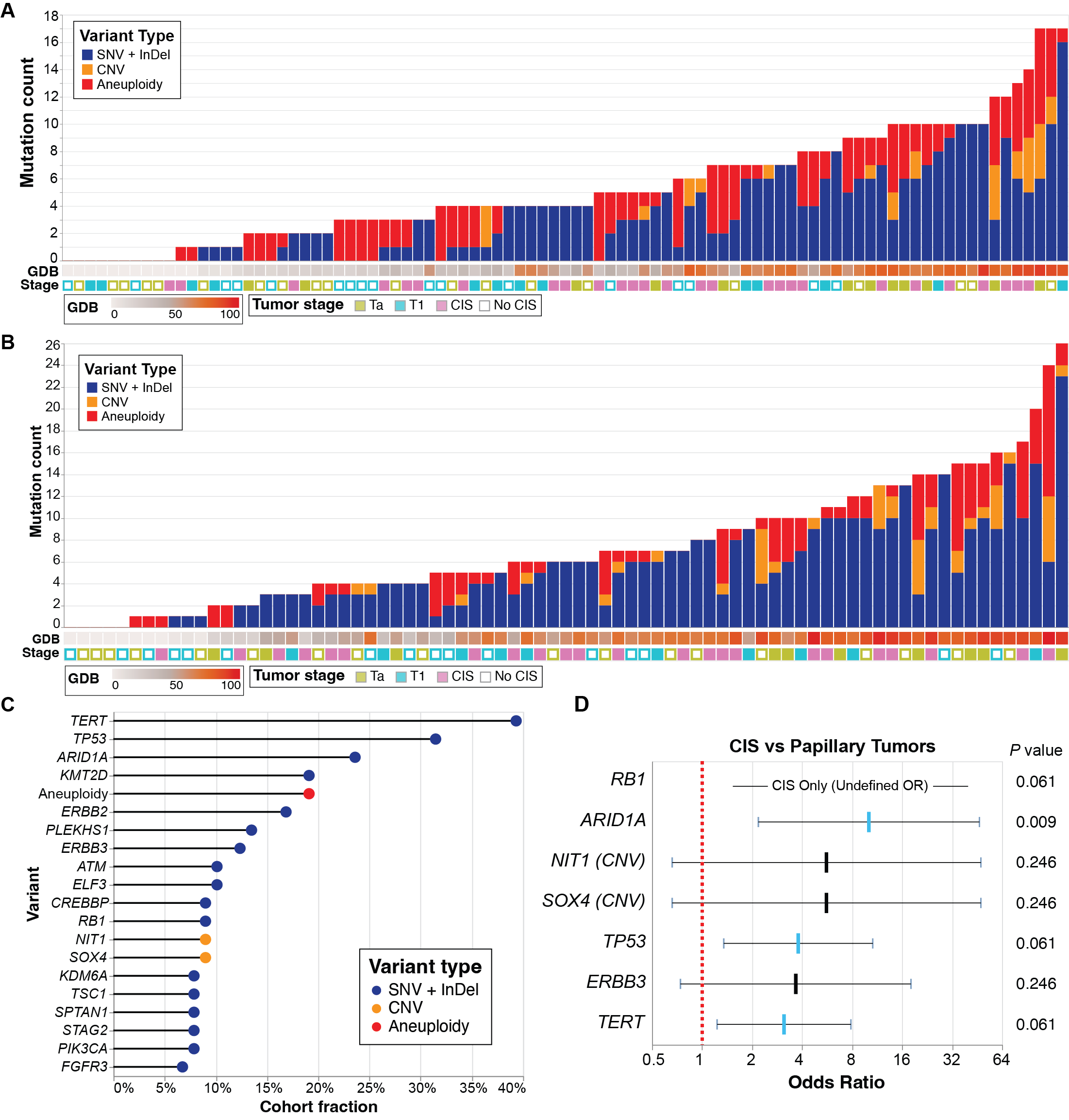

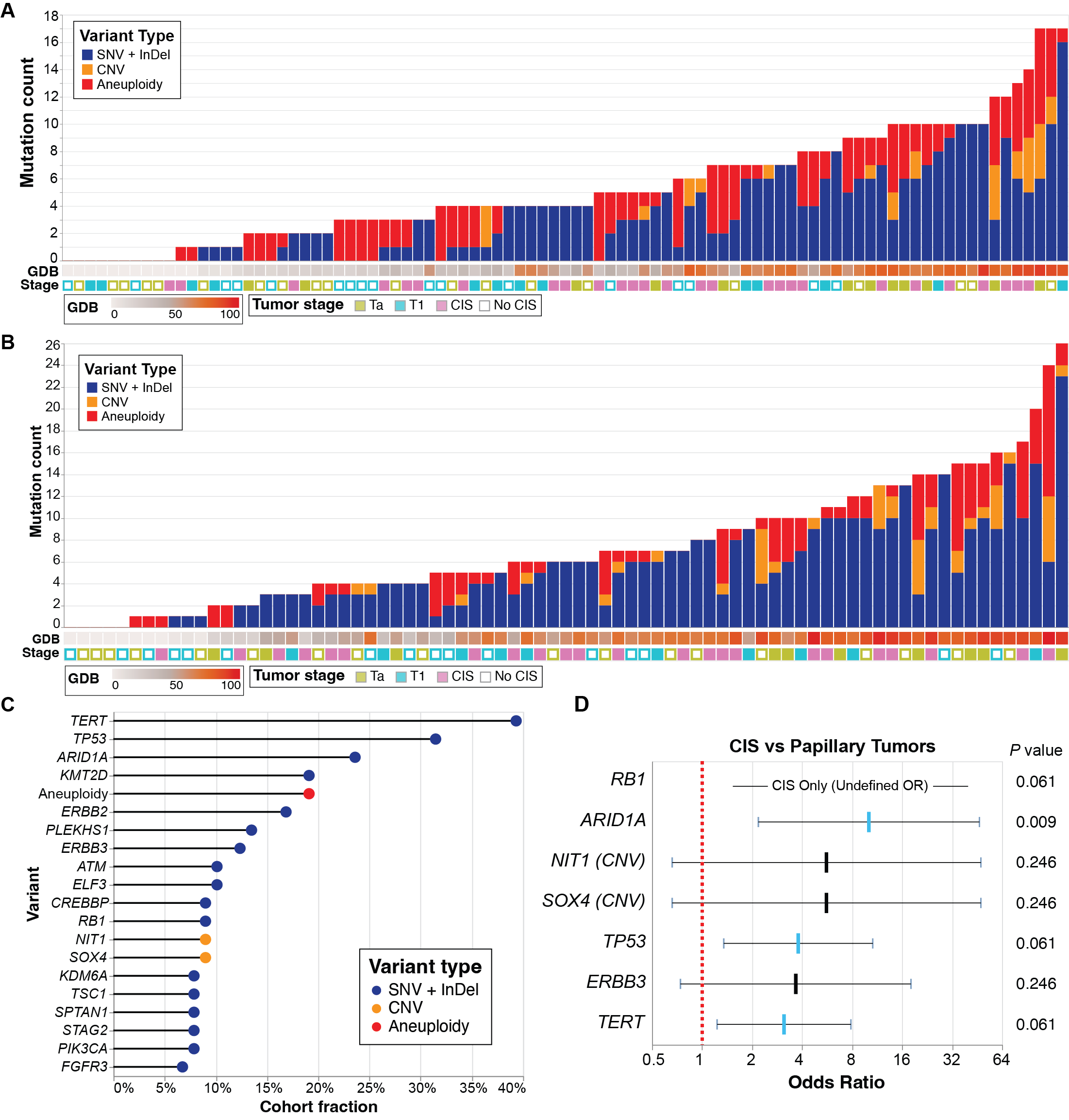


**Supplementary Fig. 3 – Genomic characterization of baseline urine samples. Profiling of utDNA was performed on urine samples from patients with BCG-unresponsive non–muscle-invasive bladder cancer. (A) Cumulative mutation counts and genomic disease burden (GDB) for specimens collected at baseline (prior to first treatment with atezolizumab; *n* = 89). (B) Oncopanel of mutation profile at study entry. Pretreatment tumor staging is indicated by color: Ta, gold; T1, blue; and CIS, pink. Solid boxes indicate the presence of CIS (either pure or concomitant). (C) Lollipop plot demonstrates the most frequent variants in the cohort. BCG = bacillus Calmette-Guérin; CIS = carcinoma in situ; CNV = copy-number variation; InDel = insertion-deletion; SNV = single-nucleotide variant; utDNA = urine tumor DNA.**


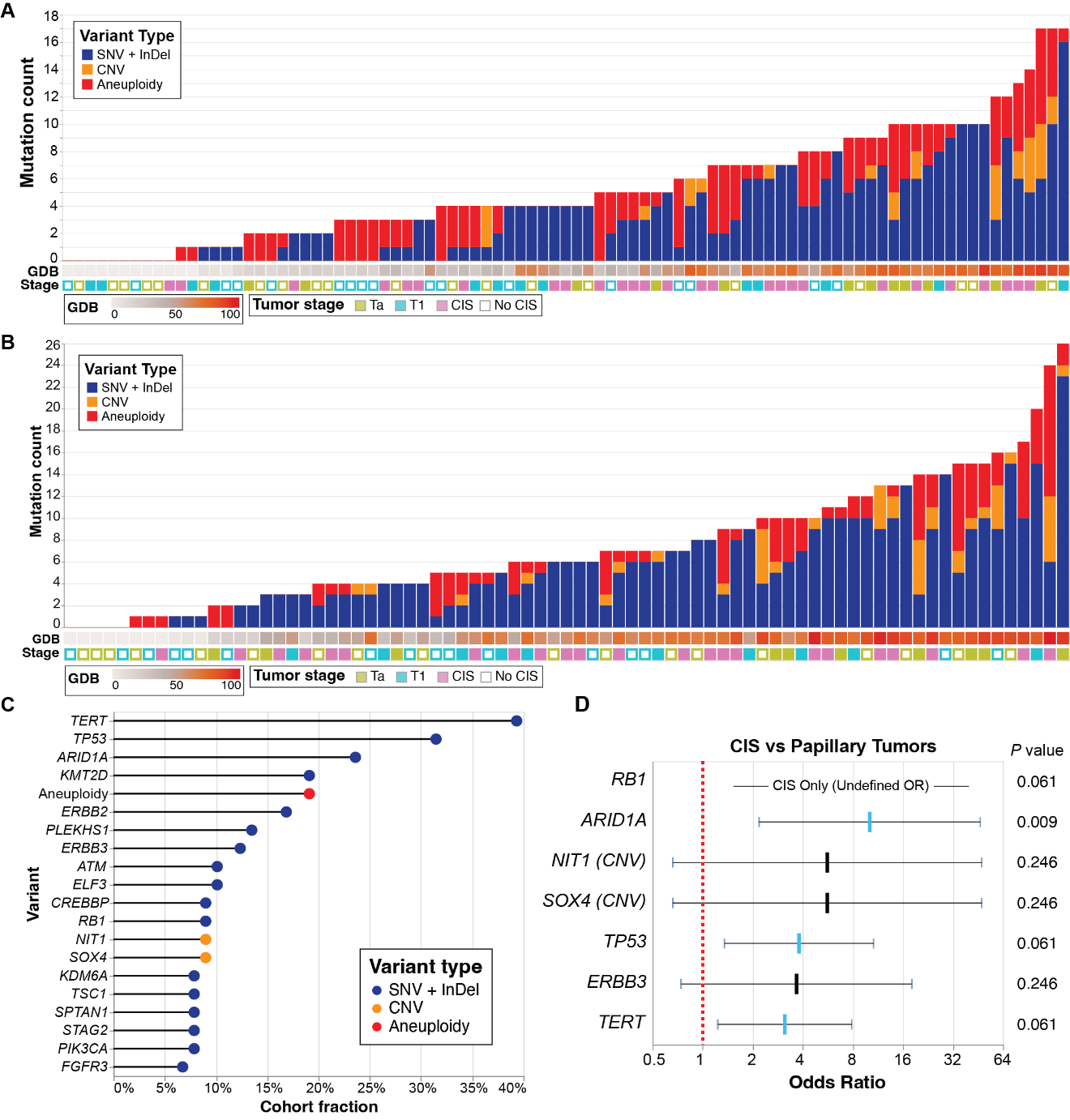

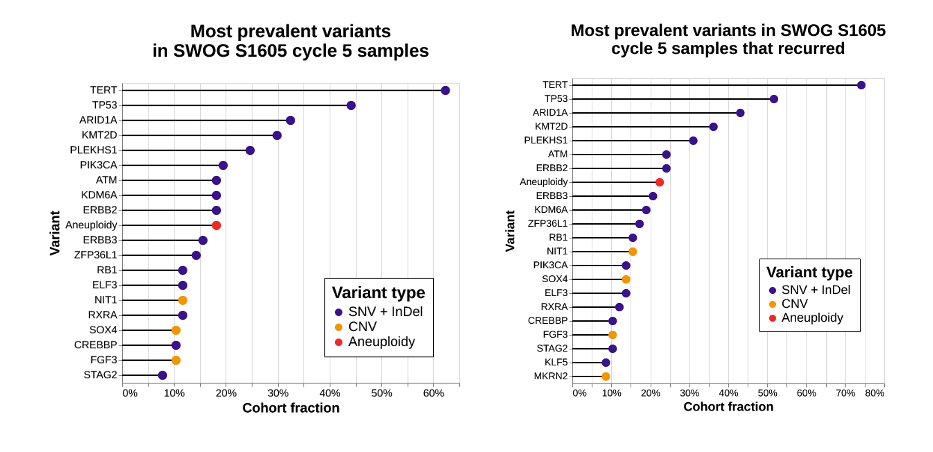

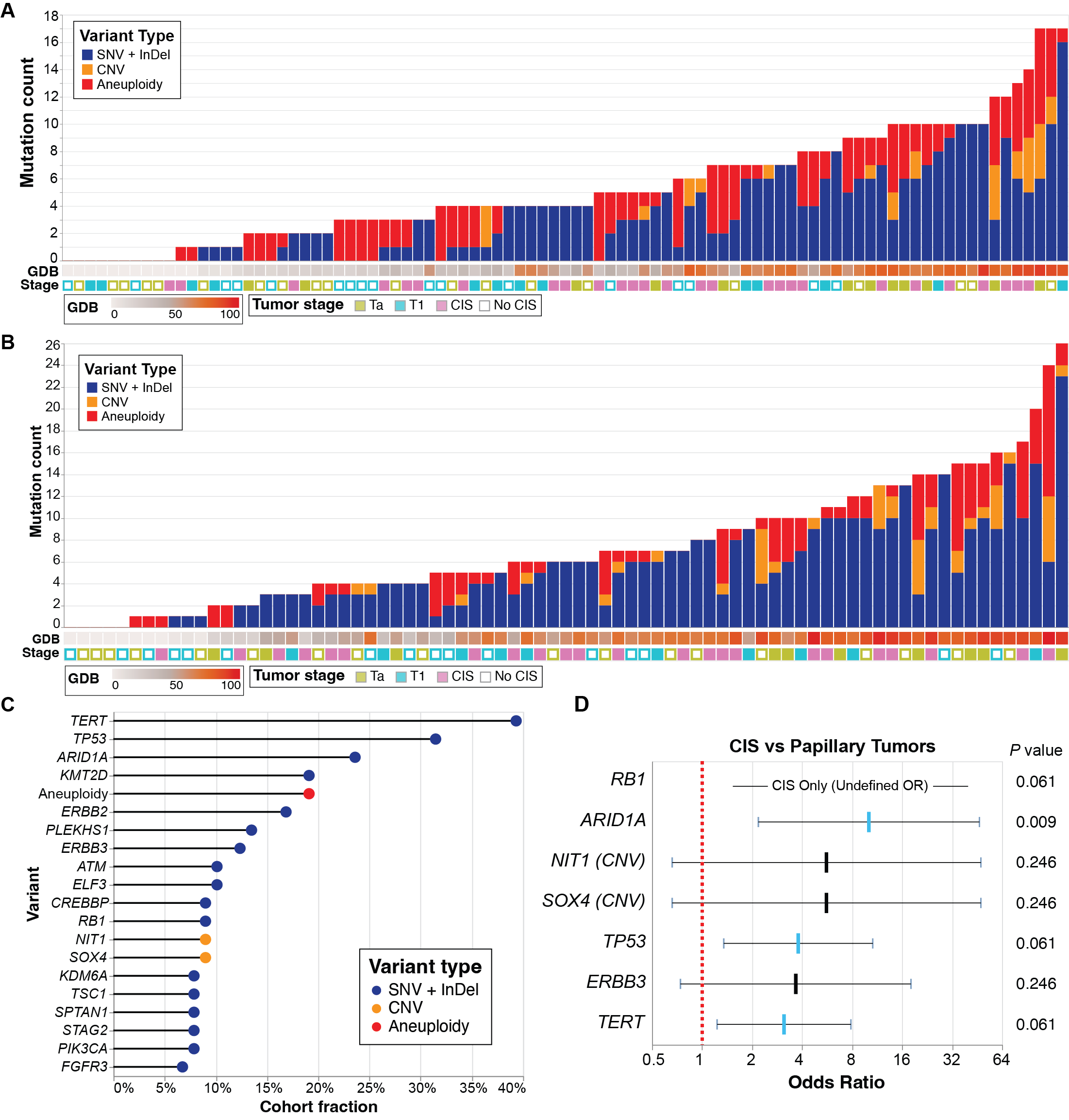

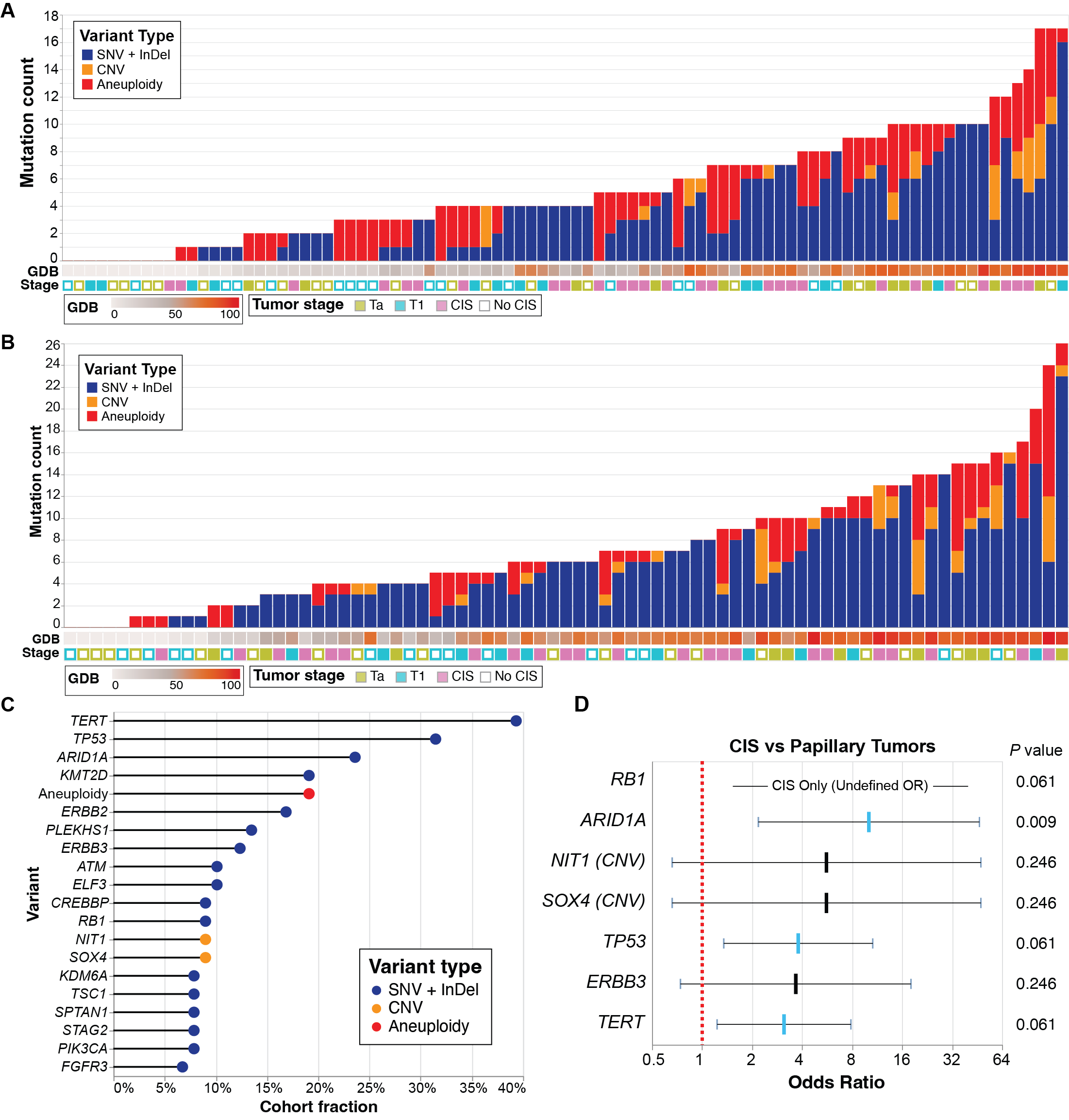

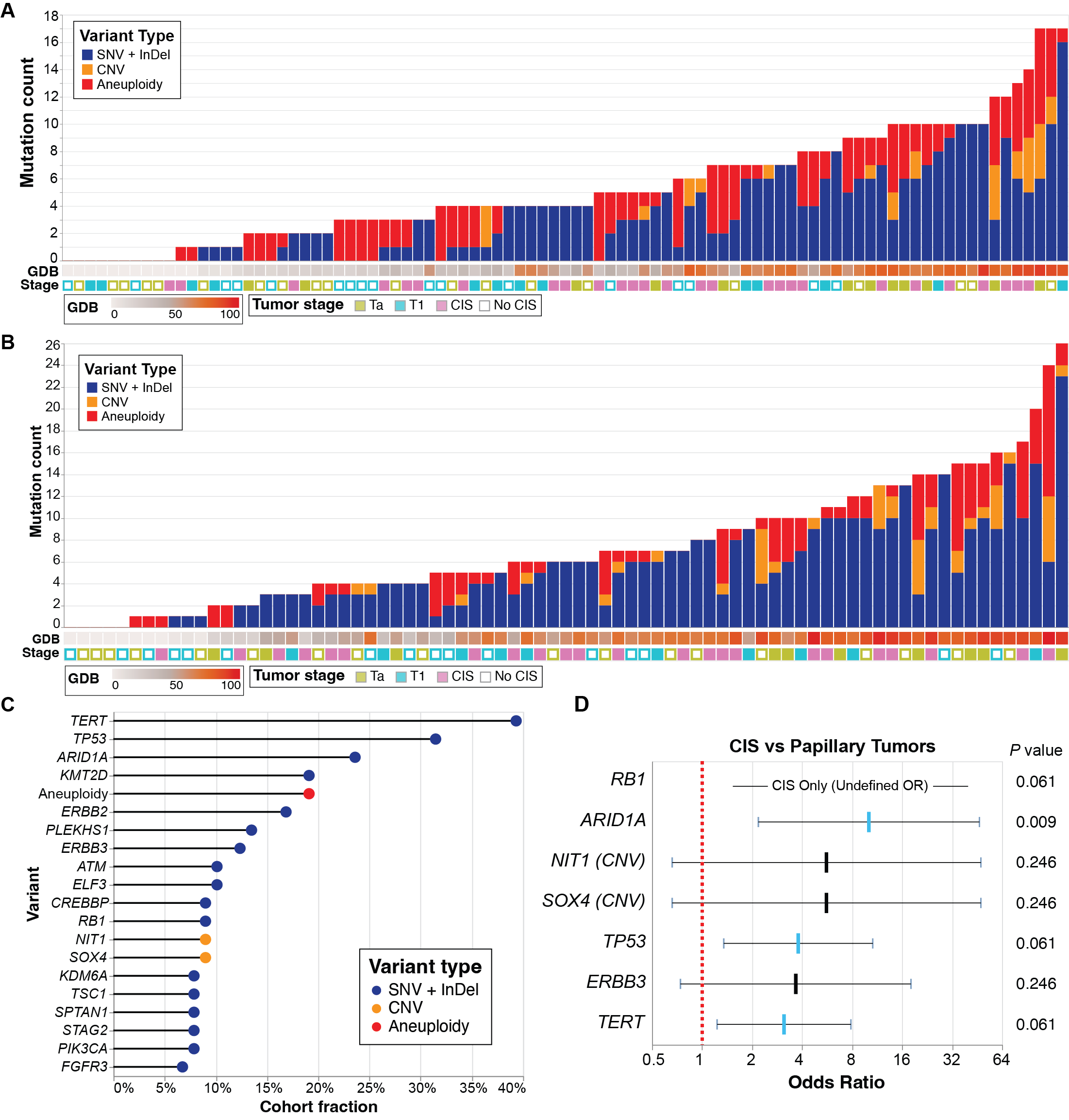


**Most prevalent mutations in 3-month samples from patients who recurred**

**Most prevalent mutations in 3-month samples from SWOG S1605 patients**

**Supplementary Fig. 4 – Genomic characterization of month 3 urine samples. Profiling of utDNA was performed on urine samples from patients with BCG-unresponsive non–muscle invasive bladder cancer. (A) Cumulative mutation counts and genomic disease burden (GDB) for specimens collected at 3 mo (day 1 of fifth cycle of atezolizumab, at the first surveillance; *n* = 77). Pretreatment tumor staging is indicated by color: Ta, gold; T1, blue; and CIS, pink. Solid boxes indicate the presence of CIS (either pure or concomitant). Summary of the most prevalent mutations (B) in all 3-mo samples and (C) from patients who recurred. BCG = bacillus Calmette-Guérin; CIS = carcinoma in situ; CNV = copy-number variation; InDel = insertion-deletion; SNV = single-nucleotide variant; utDNA = urine tumor DNA.**

**Supplementary Table 2 – Baseline results of urine cytology and UroAmp**

| Cytology result  UroAmp | Negative | Positive |
| --- | --- | --- |
| Negative | 27 | 1 |
| Positive | 51 | 10 |

**Supplementary Table 3 – Three-month results of urine cytology and UroAmp**

| Cytology result  UroAmp | Negative | Positive |
| --- | --- | --- |
| Negative | 14 | 1 |
| Positive | 52 | 8 |

**Supplementary Table 4 – Comparison of 3-mo urine cytology and UroAmp performance metrics relative to 3- and 6-mo clinical evaluation for recurrence**

|  | Recurrence at 3 mo (*n* = 75) | | Recurrence at 6 mo (*n* =75) | |
| --- | --- | --- | --- | --- |
|  | Cytology | UroAmp MRD | Cytology | UroAmp MRD |
| % Sensitivity (95% CI) ^a^ | 17 (4–41) | 100 (82–100) | 18 (8–34) | 97 (87–100) |
| % Specificity (95% CI) ^a^ | 90 (79–96) | 26 (16–40) | 94 (81–99) | 39 (23–57) |
| % PPV (95% CI) ^a^ | 33 (8–70) | 30 (19–43) | 78 (5–23) | 63 (50–75) |
| % NPV (95% CI) ^a^ | 77 (65–87) | 100 (78–100) | 52 (68–100) | 93 (68–100) |
| % Accuracy (95% CI) ^a^ | 72 (60–82) | 44 (33–56) | 55 (43–66) | 69 (58–80) |

CI = confidence interval; NPV = negative predictive value; PPV= positive predictive value.

^a^ The 95% CI values were calculated using the exact binomial method.

**Supplementary Fig. 5 – Gene mutation enrichment in carcinoma in situ (CIS). (A) Odds ratios (ORs) with 95% confidence intervals for variants enriched in BCG-unresponsive patients with CIS (*n* = 56) compared with patients with papillary-only tumors (*n* = 42) among eligible patients with available urine (*n* = 98). False discovery rate–adjusted *p* values are shown. (B) ORs with 95% confidence intervals for variants enriched in patients with CIS (*n* = 71) compared with patients with papillary-only tumors (*n* = 50) among all enrolled patients with baseline UroAmp sequencing available (*n* = 121). False discovery rate–adjusted *p* values are shown. CIS = carcinoma in situ.**

**Supplementary Fig. 6 – Event-free survival by GDB-enhanced UroAmp at 3 mo. Urinary tumor DNA (utDNA) profiling was performed after four cycles of atezolizumab at the first surveillance time point (3 mo) in patients who were not found to have a clinical recurrence (cystoscopy ± biopsy). Event-free survival was stratified by UroAmp GDB-enhanced status; Cox proportional hazards ratio was 4.3 (*p* = 0.007, 95% CI [1.5, 12.4]), adjusted for CIS status at baseline. GDB = genomic disease burden.**

**Supplementary Fig. 7 – Event-free survival (EFS) by UroAmp at baseline with revised analysis. (A) EFS by UroAmp status (negative vs positive) following clinical and genomic data adjudication. The vertical green line marks 18 mo. Hazard ratios were determined by Cox proportional hazards models with adjustment for CIS at baseline. Time 0 is the date of sample collection. (B) Cox proportional hazard ratios with 95% confidence intervals were calculated for UroAmp recurrence risk (positive vs negative) and CIS status (present vs absent). CIS = carcinoma in situ.**

**Supplementary Fig. 8 – Event-free survival by UroAmp at 3 mo of atezolizumab treatment with a revised analysis. Event-free survival stratified by UroAmp negative and positive following clinical and genomic data revision. The vertical green line marks 18 mo. Time 0 represents the time of urine collection. The hazard ratio was determined by Cox proportional hazards regression with adjustment for CIS at baseline. CIS = carcinoma in situ.**

**Supplementary Fig. 9 – Event-free survival by GDB-enhanced UroAmp at 3 mo of atezolizumab treatment with a revised analysis. Event-free survival stratified by UroAmp GDB-enhanced status (negative/positive) in 3-mo samples. The vertical green line marks 18 mo. The hazard ratio was determined by Cox proportional hazards models with adjustment for CIS at baseline. CIS = carcinoma in situ; GDB = genomic disease burden.**


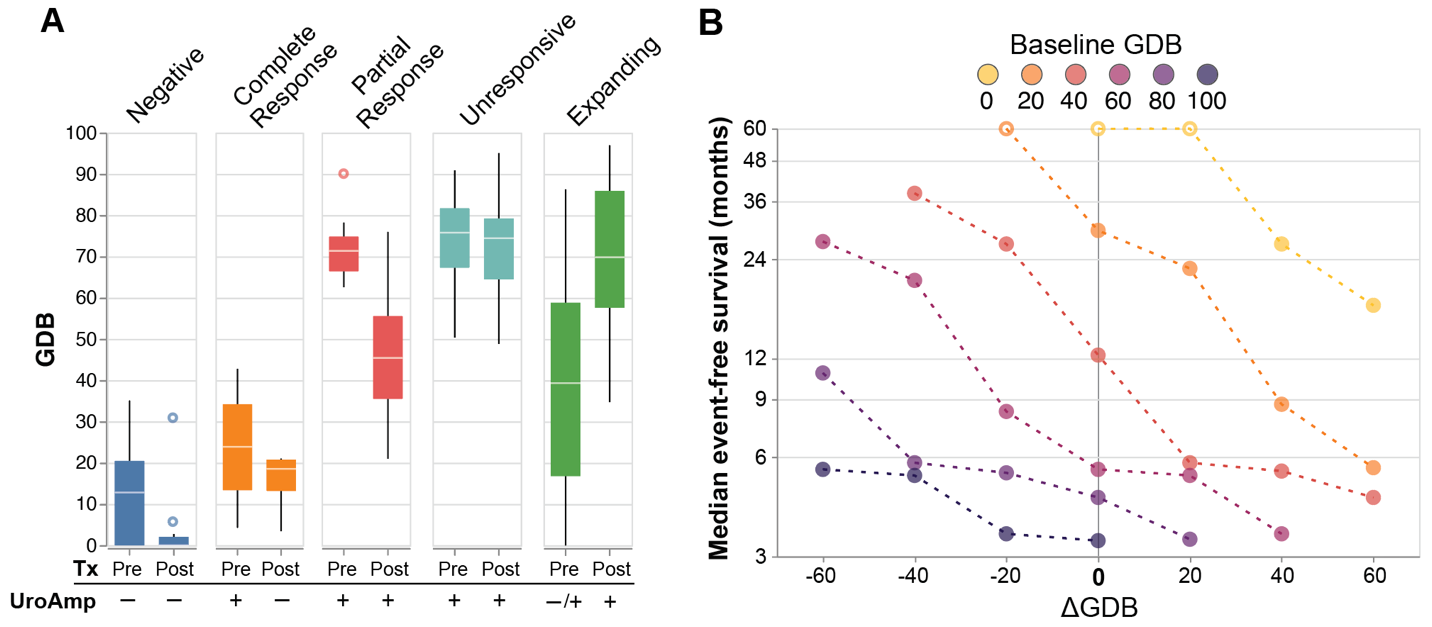


**Supplementary Fig. 10 – Longitudinal UroAmp assessment quantifies molecular response to treatment. Urinary tumor DNA profiling was performed on paired urine samples (*n* = 68) before the first and fifth cycles (3 mo) of atezolizumab treatment to measure changes in the genomic disease burden (GDB). Median event-free survival (EFS) times were estimated by the Cox survival model fitted to paired longitudinal GDB scores (baseline GDB and change in GDB). Trend lines represent estimated median EFS changes for a given baseline GDB (0, 20, 40, 60, 80, and 100 are shown) across a set of longitudinal changes in GDB (ΔGDB = 3-mo GDB minus baseline GDB), ranging from a large decrease (–60) to a large increase (+60). An open circle indicates a data point with infinite median survival (the predicted EFS curve never crosses 50% probability).**

**References**

[1] Black PC, Tangen CM, Singh P, et al. Phase 2 trial of atezolizumab in bacillus Calmette-Guerin-unresponsive high-risk non-muscle-invasive bladder cancer: SWOG S1605. Eur Urol 2023;84:536–44.

[2] US Department of Health and Human Services FDA. BCG-unresponsive nonmuscle invasive bladder cancer: developing drugs and biologics for treatment. Guidance for industry. Silver Spring, MD: FDA; 2018.

[3] Salari K, Sundi D, Lee JJ, et al. Development and multicenter case-control validation of urinary comprehensive genomic profiling for urothelial carcinoma diagnosis, surveillance, and risk prediction. Clin Cancer Res 2023;29:3668–80.

[4] Bicocca VT, Phillips KG, Fischer DS, et al. Urinary comprehensive genomic profiling correlates urothelial carcinoma mutations with clinical risk and efficacy of intervention. J Clin Med 2022;11:5827.

[5] Rac G, Patel HD, James C, et al. Urinary comprehensive genomic profiling predicts urothelial carcinoma recurrence and identifies responders to intravesical therapy. Mol Oncol 2024;18:291–304.

[6] Davidson-Pilon C. Lifelines: survival analysis in Python. J Open Source Softw 2019;4:1317.

[7] Virtanen P, Gommers R, Oliphant T, et al. SciPy 1.0: fundamental algorithms for scientific computing in Python. Nat Meth 2020;17:1–12.

[8] Seabold S, Perktold J. Statsmodels: econometric and statistical modeling with Python. Proceedings of the 9th Python in Science Conference. 2010.

[9] Chen S, Francioli LC, Goodrich JK, et al. A genomic mutational constraint map using variation in 76,156 human genomes. Nature 2024;625:92–100.
